# The Epidemiology of *Plasmodium falciparum* Malaria in the Bijagos Islands of Guinea-Bissau

**DOI:** 10.4269/ajtmh.20-1029

**Published:** 2021-03-29

**Authors:** David McGregor, Eunice Texeira da Silva, Lynn Grignard, Adriana Goncalves, Hristina Vasileva, David Mabey, Anna Last

**Affiliations:** 1 Clinical Research Department, London School of Hygiene and Tropical Medicine, London, United Kingdom;; 2 Region Sanitaria Bolama-Bijagós, Bubaque, Guinea-Bissau;; 3 Department of Infection Biology, London School of Hygiene and Tropical Medicine, London, United Kingdom

## Abstract

Distribution of long-lasting insecticide-treated nets (LLINs), passive detection and treatment with artemisinin-based combination therapy (ACT), and intermittent preventive treatment in pregnancy (IPTp) are the mainstay malaria control measures of Guinea-Bissau’s national control programme. This study aimed to estimate the prevalence of *Plasmodium falciparum* on Bubaque, the most populous island of the country’s remote Bijagos archipelago. A cross-sectional survey was performed at the start of the rainy season in August 2017. Participants were recruited using systematic random sampling in a two-stage stratified cluster design. Malaria parasitemia was detected using rapid diagnostic tests (RDTs) and quantitative PCR (qPCR). Data on housing, education, larval source management, socioeconomic status, anemia, and malaria preventive measures were collected. Multivariable logistic regression models were constructed to identify associations with *P. falciparum* infection. Four hundred four persons (aged 6 months–79 years, median 17 years) were enrolled in the study. The prevalence of *P. falciparum* parasitemia was 5.8% by RDT (95% CI: 3.55–9.33) and 16.9% by qPCR (95% CI: 13.09–21.71). The prevalence of anemia was 74.3% (95% CI: 69.04–78.85) as defined by the WHO criteria. All sampled houses were found to have open eaves; 99.5% of the surveyed population reported sleeping under a bednet (95% CI: 97.8–99.9). Although reported LLIN use is high, there remains an appreciable prevalence of malaria, suggesting that transmission is ongoing and further tools are required to reduce the burden of the disease.

## INTRODUCTION

In 2016, the West African subregion had the highest number of cases of malaria due to *Plasmodium falciparum*, with an estimated 111 million cases.^1^ Within the subregion, Guinea-Bissau reported an estimated 132,600 cases and 600 deaths from malaria that year. There is little published information on the epidemiology of *P. falciparum* infections in the country using molecular diagnostic techniques. Snounou et al.’s seminal 1993 study was an early PCR-based malaria survey during the rainy season in the village of Bor, west of Bissau, the country’s capital city. Of 79 asymptomatic persons (all ages) surveyed, 69 persons were reportedly infected with *Plasmodium* species, including 49 with *P. falciparum*, a positivity rate of 87%. A study by Santoguina et al.^2^ in 2009 of 2,348 asymptomatic persons (all ages) at three sites in the Gambia and Guinea-Bissau (West of Bissau), respectively, found an overall parasite prevalence of 25.5% (95% CIs: 23.7–27.3%). All three sites had comparable prevalence of *P. falciparum* infections.

The epidemiology of *P. falciparum* in the country’s offshore Bolama Bijagos region has not previously been reported. The region’s approximate population of 30,000 lives on 20 different islands in the archipelago. The region has its own hospital, which includes a diagnostic laboratory, on the island of Bubaque. However, the remoteness of the islands from the mainland, and the distance between villages, impedes the collection of accurate epidemiological data. Other African islands have previously reported significant success in reducing malaria prevalence. Scaling up vector control programs on the islands of the Comoros,^3^ Zanzibar,^4^ and São Tomé and Príncipe^5^ have been credited for this. Mass drug administration with antimalarial drugs also offers the possibility of reducing prevalence on islands significantly and durably.^6^ To assess the susceptibility of the archipelago to such strategies, we conducted the first cross-sectional population-based malaria survey in the Bolama Bijagos region. The island of Bubaque was the chosen site for this malaria survey. It is situated approximately 50 km to the southwest of Bissau. The island has a tropical climate and a rainy season from June to October. A once to twice weekly shuttle boat connects the island to Bissau. A qualitative study by Durrans et al.^7^ demonstrated that travel patterns between islands and the mainland are complex and fluctuate according to seasons.

The island survey allowed us to collect detailed clinical and epidemiological data including reported use of long-lasting insecticide-treated nets (LLINs), uptake of intermittent preventive treatment in pregnancy (IPTp), prevalence and severity of anemia, and prevalence of *P. falciparum* parasitemia as measured by rapid diagnostic test (RDT) and quantitative PCR (qPCR). Asymptomatic parasitemic individuals are the largest reservoir of *P. falciparum*, and estimating the size of this group in the population was the primary objective of this study.^8^

## MATERIALS AND METHODS

### Census.

A census of the island was carried out in July 2017 to provide a sampling frame of households and an updated estimate of the island population. A cross-sectional population-based survey was carried out in August of that year.

### Study design and randomization.

A malariometric cross-sectional survey was conducted using RDTs and qPCR on a representative group of asymptomatic individuals. A stratified cluster sampling approach was used to account for the stratification of individuals by village and clustering by households (primary sampling units). Probability proportional to size sampling was used in the island’s 14 villages. Households were selected by simple random selection using household census Global Positioning System coordinates. All asymptomatic household members aged 6 months and older were invited to join the study.

### Survey.

Participants were interviewed using a standardized structured questionnaire. For participants younger than 7 years, the interview was completed with the main caregiver. Answers were recorded on tablets using Open Data Kit (ODK, Get ODK Inc., San Diego, CA) and uploaded to a secure and encrypted server. Demographic information such as age, gender, education level, pregnancy status, and bednet use was recorded. Further household information was collected as guided by the Household Survey Indicators for Malaria Control and the Malaria Indicator Survey. This included questions on the level of education, material possessions, housing quality, treatment-seeking behavior, and vector control measures.

### Sample collection and storage.

Blood from each participant was obtained by finger prick sampling using a standard procedure onto Whatman 3MM filter paper and used for RDT (Carestart Malaria, AccessBio, Somerset, NJ) and point-of-care hemoglobin measurement (Hemocue, Ängelholm, Sweden). Approximately 20 μL of whole blood was collected onto filter paper from each participant, air dried, stored at −20°C, and shipped within a fortnight of collection to the molecular laboratory at the London School of Hygiene and Tropical Medicine, London, United Kingdom.

### Rapid diagnostic tests.

Approximately 5 μL of whole blood was obtained from participants to detect *Plasmodium* species using a CareStart Malaria histidine-rich protein 2 (HRP2)/parasite lactate dehydrogenase (pLDH) (Pan) Combo RDT (ACCESS BIO, NJ). The presence of HRP2 and lactose dehydrogenase in the blood identifies *P. falciparum*.

### Elution, DNA extraction, and qPCR.

DNA was extracted from dried blood samples collected onto a filter paper. Blood spots were punched with a sterile hole puncher. DNA was extracted using a PureLink Pro 96 well Genomic DNA kit following the manufacturer’s instructions (Invitrogen, Carlsbad, CA). *Plasmodium falciparum* species–specific qPCR was performed in duplicates, using 5 µL of samples DNA, forward (5′-GTAATT GGA ATG ATA GGA ATT TAC AAG GT-3′) and reverse (5′-TCA artemisinin-based combination therapy (ACT) ACG AAC GTT TTA ACT GCA AC-3′) primers, CYS5-FAM probe, and TaqMan™ Multiplex Master Mix (Thermo Fisher Scientific, Waltham, MA). Quantitative PCR was performed in 96-well plate on the AB 7500 FAST system (Applied Biosystems, Foster City, CA). The first WHO international standard for nucleic-acid amplification test (NAT) analysis was used as qPCR control in a form of a standard curve.^9^

### Statistical analysis.

Rapid diagnostic tests, qPCR, and survey questionnaire results were checked for completeness and analyzed in Stata v. 15 (SataCorp, College Station, TX). Survey module settings were adjusted for the stratified cluster design with finite population corrections. The Shapiro–Wilk test was used as the test of normality. Student’s *t*-test, Pearson’s chi-squared test, and Fisher’s exact test were used as appropriate. A generalized linear model forward univariable logistic regression was used to identify associations between *P. falciparum* parasitemia and questionnaire variables. Final multivariable logistic regression models were built using variables of interest identified a priori (age and education) and those which were significantly linked to the outcome in the sequential analyses to adjust for potential confounders. *P*-values less than 0.05 were considered statistically significant.

### Ethical considerations.

Ethical approval for the study was obtained from the Comite Nacional de Eticas de Saude in Guinea Bissau and from the London School of Hygiene and Tropical Medicine Ethics Committee. Written informed consent or adult caregiver consent in lieu when younger than 18 years was required. Written assent for participants aged 7–17 years was additionally required.

## RESULTS

**Figure 1.**
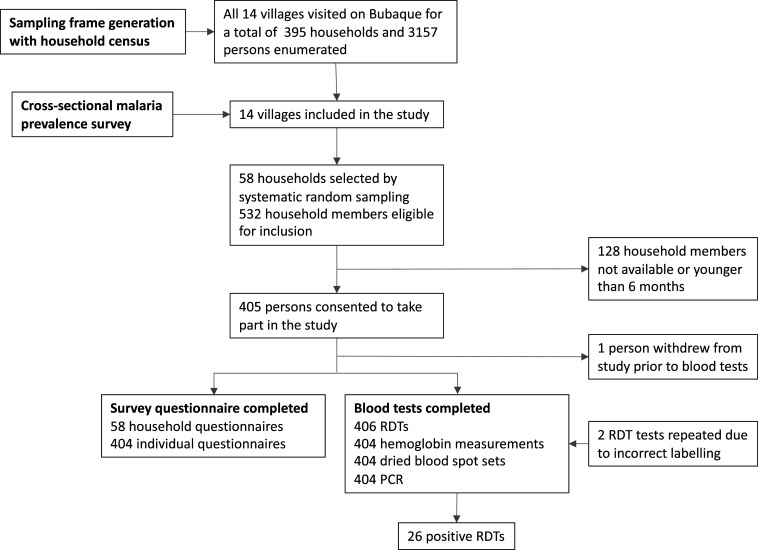
Study flowchart.

Four hundred four individuals from 58 households took part in the survey (Figure 1). Characteristics of participants, including age-adjusted prevalence data, and households are presented in Table 1 and Supplemental Table 3, respectively. The level of formal education achieved—primary, secondary, and higher—was comparable across genders (Table 1), but males had on average 4.5 years of education (95% CI: 4–5) compared with 3.3 years for females (95% CI: 3–4). The most educated person in each household had on average 7.7 years of formal schooling (95% CI: 6.6–8.8) (Supplemental Table 3); 48.3% of households had a female person as their most educated member; such households had a similar proportion of *P. falciparum* cases as households in which the most educated person was male (*P* = 0.99). The mean hemoglobin level across all surveyed individuals was 110.6 g/dL (95% CI: 108.3–112.8). More than a quarter of participants had a normal hemoglobin level for their gender and age as defined by the WHO criteria (25.7%, 95% CI: 21.1–31) (Supplemental Table 6).^10^ Women were more likely to be anemic than men, according to the WHO age-adjusted criteria, with an average of 107 g/dL (95% CI: 105–109) versus 113 g/dL (95% CI: 111–117), respectively (*P* = 0.0006).

**Table 1:** Demographic data of individuals

*n* = 404	Value	95% CI
Mean age (years)	21	19–24
Population sampled by age-groups (years) (%)		
< 5	25.1	20.1–30.8
6–15	21.4	17.4–26.1
16–30	25.9	22.7–29.4
≥ 31	27.5	29.5–33.2
Gender (% female)	58.1	52.8–63.4
Mosquito net used last night (%)	99.5	97.8–99.9
School education level (%)		
Nil	51.1	46.3–56
Primary	29.7	25.7–34.2
Secondary	18.8	15–23.4
Higher education	0.25	0–1.9

**Figure 2.**
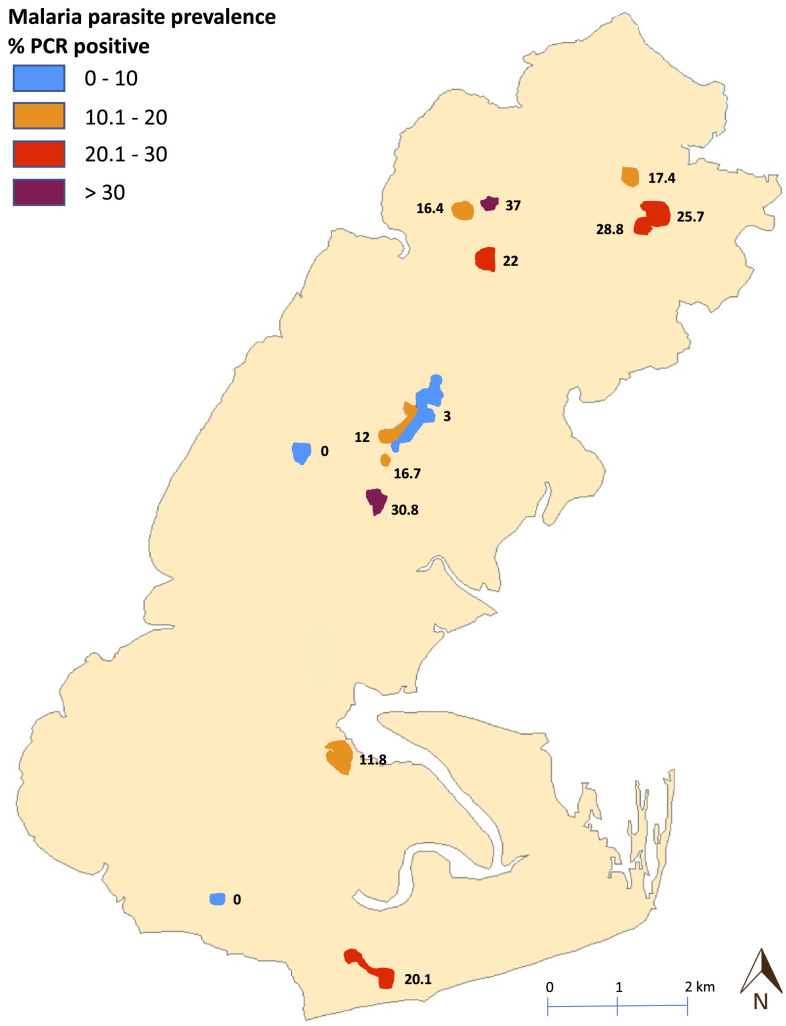
Spatial distribution of parasite prevalence.This figure appears in color at www.ajtmh.org.

The overall estimated prevalence was 5.8% (95% CI: 3.5–9.3) by RDT (26/404) and 16.9% (95% CI: 13.1–21.7) by qPCR (75/404). Village prevalence rates ranged from 0% to 25% by RDT and 0–37% by qPCR (Figure 2). Participants between the ages of 6 and 15 years had highest prevalence of *P. falciparum* parasitemia with 25.6% compared with 14.9% for participants younger than 5 years, 18.7% for 16–30-year olds, and 15.9% for those older than 30 years (Table 2). Using qPCR as the reference method, RDT sensitivity was 84.6% (95% CI: 65.1–95.6) and specificity 86.0% (95% CI: 82.1–89.3%). The mean qPCR cycle threshold (CT) difference for qPCR-positive cases according to their RDT result was 30.4 (95% CI: 28.6–32.2) for RDT-positive cases compared with 34.4 (95% CI: 33.7–35.2) for RDT-negative cases (*P* < 0.001) (Supplemental Figure 4).

**Table 2:** Individual factors associated with *Plasmodium falciparum* parasitemia by PCR–multivariate analysis

Factor	Total (*n* = 404)	PCR positive (%)	Odds Ratio	95% CI	*P*-value
Gender					
Female	238	43 (18.1)	1.07	0.527–2.17	0.846
Male	166	32 (19.3)	1		
Age-groups (years)					
< 5	80	14 (14.9)	1	–	–
6–15	67	23 (25.6)	3.08	1.09–8.69	**0.034**
16–30	87	20 (18.7)	1.46	0.51–4.15	0.461
≥ 31	95	18 (15.9)	1.38	0.50–3.81	0.520
Years of formal education					
0	171	34 (16.6)	1	–	–
1–6	102	23 (18.4)	0.84	0.32–2.17	0.718
7–12	55	18 (24.7)	1.64	0.58–4.64	0.342
12 and older	1	0 (0)	Omitted	–	–
Anemia					
No anemia	17	6 (35.3)	4.01	1.44–11.17	**0.009**
Mild	152	25 (16.4)	1.06	0.512–2.22	0.856
Moderate	131	26 (19.8)	1.61	0.638–4.08	0.304
Severe	102	16 (15.7)	1	–	–

Bold figure shows significance at *P* < 0.05.

An estimated 68.4% (20/30) of women pregnant in the previous 2 years reported having received a full course of IPTp (sulfadoxine with pyrimethamine) during their pregnancy (95% CI: 46.3–84.5) (Supplemental Table 5); 99.5% (402/404; 95% CI: 97.8–99.9) of participants reported sleeping under a mosquito net the previous night. All surveyed houses had open eaves, and none used indoor residual spraying (IRS); 15% of household heads scored three of three for questions on mosquito larva knowledge and management (95% CI: 7.7–27.1); however, this was not associated with a reduced probability of someone in the household testing positive (Supplemental Table 4). Regarding diagnosis and treatment, 14 children younger than 5 years were reported as having had a fever in the previous 2 weeks; 63.5% (10/14) of such children were tested either by microscopy or RDT by a local healthcare worker (95% CI: 32–95). Antimalarials were given to an estimated 56.9% (9/14) of these children (95% CI: 25.9–87.8), all first line (artemether with lumefantrine).

## DISCUSSION

The primary objective of this cross-sectional survey was to obtain a rainy season estimate of the prevalence of *P. falciparum* parasitemia on Bubaque, which was found to be 16.9% (95% CI: 13.1–21.7) using qPCR. The survey was completed early in the rainy season (August), and so prevalence numbers may have been lower than if assessed a few months later (November) as observed in a neighboring country with similar malaria endemicity.^11^ Marked differences in estimated prevalence between villages were observed from 0% to 37% by qPCR. Such differences may reflect the limited number of samples obtained in smaller villages. Local circumstances and practices may also be at play to increase parasite exposure. Some of the highest prevalence numbers were found in village populations which resettled in temporary shelters next to their crop fields during the rainy season to facilitate the harvest. Despite high LLIN usage, such migratory practices may increase parasite exposure.

Elsewhere in Guinea-Bissau, the most recent survey, conducted in the coastal area of Caio in 2009, reported a prevalence of 4% with microscopy (95% CI: 2.6–5.4) and 23.7% with qPCR (95% CI: 20.9–26.7) (all ages).^2^ Previous studies in the 1980s and 1990s, using light microscopy as the diagnostic tool, reported prevalence estimates ranging from 21%^12^ (all ages) to 79% in children (2–9 years of age) in northwestern Guinea-Bissau,^13^ and 28% in the west of country.^14^ A study focusing on children aged 3–6 years in the capital city of Bissau in 1994 reported a prevalence of 59%,^15^ and another in 1999 reported a prevalence of 26% in children aged 1–2 years^16^; a study of children and adults in 2008 found a prevalence of just 3% at the end of the rainy season.^17^ The heterogeneity of eligibility criteria, seasonal timing, and diagnostic procedure across surveys make direct comparisons of results difficult. This problem is well documented in many sub-Saharan countries and is not limited to Guinea-Bissau.^18^ However, the trend across these surveys suggests a stable or slightly decreasing prevalence over the past 20 years in Guinea-Bissau.

The secondary objective of this study was to investigate associations between demographic, nutritional, educational, and socioeconomic indicators, and malaria parasitemia on Bubaque. In this study, 6–15-year-olds were found to have the highest prevalence of *P. falciparum* parasitemia by qPCR. In the rural region of Caio, Satoguina et al.^2^ also showed that qPCR-determined parasitemia was most common in adolescence, specifically among 11–15-year-olds, and then decreased in adulthood. Malaria parasitemia and malnutrition, with anemia as a cofactor, are among the major causes of childhood morbidity and mortality in sub-Saharan Africa.^19,20^ Thorne et al.^21^ surveyed 872 children in a cross-sectional study of younger than 5 year olds on three islands of the Bijagos archipelago and reported that 80.2% of children were anemic in 2013. This converges with the current study which found that 79.9% of children in that age-group are anemic. The interplay among anemia, malaria, other parasitic infection such as soil-transmitted helminths, and malnutrition is complex.^22–25^ Early antibody response in children may also play a role resulting in severe malaria outcomes.^26^ Socioeconomic status (SES) was not found to be associated with parasitemia. Previous studies consistently associate lower SES with high infection rates.^27,28^ The ubiquity of open eaves in island houses and the absence of stark wealth inequality among households are possible mitigating factors. Education, and maternal education in particular, has been reported as being a protective factor against child malaria infection.^29–31^ In this study, however, no significant association was found among the level of household education, the gender of the most educated household member, and malaria parasitemia. This study showed that all children younger than 5 years reported having slept under a mosquito net the previous night. This compares favorably to the results of Malaria Indicator Surveys conducted in other sub-Saharan countries between 2008 and 2015.^32^ Of note, self-reporting of LLIN usage can be unreliable, and on-site checks for usage are preferred when possible.

The efficacy of LLINs in preventing malaria infection and mortality has been well documented in sub-Saharan Africa.^33,34^ In this study, nearly all persons reported sleeping under a mosquito net the previous night. Under the national malaria prevention program, LLIN distributions occur every 2–3 years. The benefits of more frequent distributions on parasite prevalence are uncertain. In a 2003 cross-sectional study of the capital city of Bissau, Rodrigues et al.^17^ reported that bednets were used regularly by 79% of respondents and increased to 92% by 2005. By comparison, the WHO^1^ estimates that, in sub-Saharan African countries as a whole, just 53% of the populations at risk slept under an LLIN in 2015 (95% CI: 50–57). A recent entomology study has reported that *Anopheles gambiae* was the main malaria vector on Bubaque and that adult mosquitoes showed full susceptibility to permethrin but moderate resistance to α-cypermethrin.^35^ A moderate frequency of the West African kdr pyrethroid conferring resistance allele was also observed. Expansion of such resistance traits in the mosquito population could compromise current control methods that rely on LLINs.

Stable climatic conditions^17,36^ and the introduction of malaria control measures, such as good case management using both RDTs and ACT, may have contributed to decreased prevalence.^37^ Community-wide free distributions of LLINs, as observed before the survey, is another effective means in reducing malaria prevalence.^38–40^ Aside from IPTp, other control measures including chemoprevention and IRS have not been widely used in Bubaque or in other regions of Guinea-Bissau. As prevalence of the parasite and the size of its human reservoir of infection decrease, it becomes increasingly challenging to diagnose subclinical parasitemia to interrupt forward transmission. The need for highly sensitive molecular diagnostics is therefore ever increasing.

## CONCLUSION

Asymptomatic *P. falciparum* infections were identified with a combination of RDT and qPCR. In this first survey in the Bijagos Archipelago of Guinea-Bissau, a wide disparity in burden of subclinical *P. falciparum* infections was found at the village level on the island of Bubaque, with a moderate prevalence overall. Such levels of subclinical parasitemia were found despite regular LLIN distributions and high reported use by participants. This suggests that novel methods may be required, in addition to the existing malaria control tool kit, to reduce and ultimately stop transmission. Further studies are needed to better understand the fine scale transmission of the parasite among villages and islands.

## Supplemental tables and figure

Supplemental materials
