# Supplementary material for: The Epidemiology of Plasmodium falciparum Malaria in the Bijagos Islands of Guinea-Bissau
Source: Am J Trop Med Hyg. 2021 Mar 29;104(6):2117–22. doi: 10.4269/ajtmh.20-1029 (PMC8176492; doi:10.4269/ajtmh.20-1029)
Supplement: Supplementary file 1 [file tpmd201029.SD1.pdf]

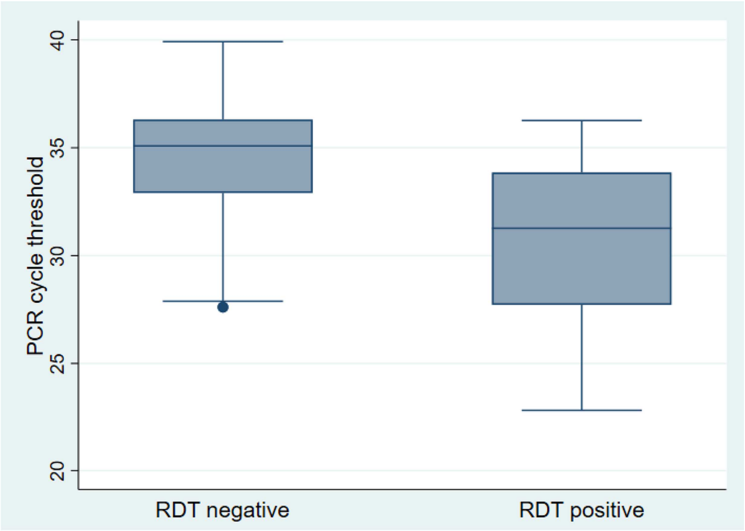

Supplementary Figure 3. QPCR cycle thresholds of *P. falciparum* cases by RDT result

Supplementary Table 3. Demographic data of households

n=58	Value	95% CI
Mean number of members per household	9.35	8.28 - 10.42
Mean number of participants per household	8.39	6.97 - 9.80
Mean school years for most educated household member	7.75	7.02 - 8.48
Most educated household member (%)		
Male	51.68	36.97 – 66.10
Female	48.31	38.89 – 63.02
Mean number of bed nets per person	0.62	0.55 – 0.69
Larval source management knowledge score		
0 correct answers	66.02	52.18 – 77.58
1 correct answer	17.63	9.43 – 30.54
2 correct answers	1.30	0.17 – 9.05
3 correct answers (max score)	15.02	7.73 – 27.17
Household residence with open eaves (%)	100	-

Supplementary Table 4. Household factors associated with Plasmodium parasitemia by PCR – multivariate analysis

n=58	OR	95% CI	p value
Household education level			
No education	-0.94	-5.64 – 3.77	0.696
Primary education (1-6 years)	1	-	
Secondary education (7-12 years)	-0.43	-1.92 – 1.07	0.574
Fever in the past 2 weeks (under 5s only)	1.6	-1.67 – 4.87	0.337
SES			
1 (lowest)	2.78	-0.41 – 5.97	0.088
2	-0.41	-2.35 – 1.52	0.675
3	0.37	-1.33 – 2.09	0.665
4	2.52	-0.8 – 5.84	0.137
5	1	-	-
Household LSM score ≥3	-0.99	-3.24 – 1.24	0.383
Household with ≥1 net per 2 persons	0.24	-1.5 – 1.98	0.787

Supplementary Table 5. Female participants

n=238	Value	p value
Education level compared to males (%)		
0 years	60.5	0.2
In primary education	63.2	0.9
In secondary education	47.9	3.6
In higher education	0	1.4
		**95% CI**
Pregnancy (as % of 15-49 year olds)		
Pregnant	3.13	1.25 – 7.63
Pregnant in past 2 years	28.53	21.37 – 36.97
ITPP received in last pregnancy		
No ITPP received (%)	31.61	15.53 – 53.73
1-2 ITPP dose (%)	0	-
≥3 ITPP doses (%)	68.39	46.26 – 84.46

Supplementary Table 6. Population estimates of anemia

	Mean	95% CI
Mean Hemoglobin level (g/dl)	110.6	108.3 – 112.8
**Adults, children and adolescents**		
Normal	25.75	21.15 – 30.96
Mild anemia	33.26	28.05 – 38.90
Moderate anemia	37.58	31.06 – 44.58
Severe anemia	3.39	1.93 – 5.91
**Adults**		
Women		
Normal	26.60	18.79 – 36.22
Mild	34.55	26.38 – 43.75
Moderate	34.63	25.67 – 44.83
Severe	4.19	1.57 – 10.68
Men		
Normal	38.76	28.45 – 50.2
Mild	49.06	36.75 – 61.47
Moderate	10.06	5.09 – 18.91
Severe	2.10	0.047 – 8.93
**Children and adolescents**		
Children 1-5 years old		
Normal	20.11	12.80 – 30.16
Mild	33.38	20.61 – 49.15
Moderate	42.93	29.82 – 57.12
Severe	3.56	1.12 – 10.74
Children 6-11 years old		
Normal	15.02	8.74 – 24.60
Mild	15.19	8.56 – 25.52
Moderate	65.33	51.56 – 76.94
Severe	4.44	1.86 – 10.21
Children 12-14 years old		
Normal	24.75	9.62 – 50.41
Mild	29.92	13.67 – 53.52
Moderate	45.31	23.96 – 68.54
Severe	0	-
